# iLDA‐SGCN: Identifying Associations Between Age‐Related Diseases and Long Non‐Coding RNAs Using Dual Graph Convolutional Networks

**DOI:** 10.1111/acel.70572

**Published:** 2026-06-09

**Authors:** Yu Guo, Shizheng Qiu, Zhishuai Zhang, Jirui Guo, Haozheng Liang, Huanyu You, Fengjuan Lu, Yanwei Xu, Yang Hu

**Affiliations:** ^1^ Center for Bioinformatics, Faculty of Computing Harbin Institute of Technology Harbin China; ^2^ Key Laboratory of Biological Bigdata, Ministry of Education Harbin Institute of Technology Harbin China; ^3^ Beidahuang Industry Group General Hospital Harbin China; ^4^ Beidahuang Group Neuropsychiatric Prevention and Treatment Hospital Harbin China

**Keywords:** age‐related diseases, aging, dual GCNs, hypertension, lncRNA marker

## Abstract

Aging reshapes global disease burdens, yet the regulatory roles of long non‐coding RNAs (lncRNAs) in age‐related disorders remain incompletely characterized. We developed iLDA‐SGCN, a graph‐based computational framework that integrates singular value decomposition (SVD) with dual graph convolutional networks (GCNs) to predict lncRNA‐disease associations. SVD first derives compact low‐dimensional representations from the lncRNA‐disease association matrix. Two complementary GCN modules then learn topology‐aware embeddings: a correlation‐map GCN operating on the bipartite lncRNA‐disease network, and a similarity‐map GCN operating on fused homogeneous graphs of lncRNAs and diseases constructed from MeSH semantic similarity and Gaussian association‐profile kernels. Finally, association scores are estimated with an inner‐product decoder optimized with a class‐imbalance‐aware loss function. Across five‐fold cross‐validation on LncRNADisease and MNDR datasets, iLDA‐SGCN outperformed five competitive methods (SDLDA, LDNFSGB, IPCARF, LDASR, and LDA‐VGHB) in terms of AUC (area under the ROC curve) and AUPR (area under the precision‐recall curve). The model achieved AUC/AUPR of 0.960/0.968 on MNDR and 0.896/0.901 on LncRNADisease, with only a marginal precision shortfall versus LDA‐VGHB on LncRNADisease. Ablation studies showed both GCN modules improved over a fully connected backbone, with the similarity‐map GCN contributing the largest gains; the full model performed best overall. In case studies across eight prototypical age‐related diseases, iLDA‐SGCN identified *HOTAIR*, *MALAT1*, *PVT1*, *MEG3*, *H19*, *LSINCT5*, *UCA1*, and other candidates, yielding 33 candidates potentially involved in age‐related disease mechanisms that require further experimental validation. Collectively, iLDA‐SGCN integrates semantic and topological information to prioritize candidate lncRNA–disease associations related to aging, providing testable hypotheses for downstream mechanistic studies.

## Graphical abstract

iLDA‐SGCN integrates singular value decomposition and dual graph convolutional networks to predict lncRNA‐disease associations. It achieves superior performance on two benchmark datasets and identifies 33 candidate lncRNAs across 8 age‐related diseases.

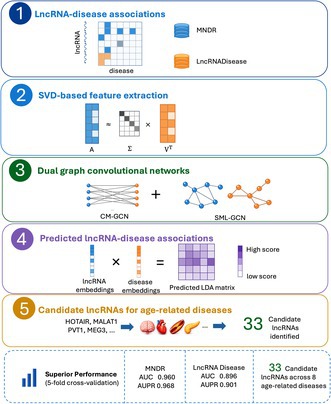

**Abbreviations**
AUC: area under the receiver operating characteristic curve
AUPR: area under the precision‐recall curve
CM‐GCN: correlation‐map graph convolutional network
GAP: gaussian association profile
GCN: graph convolutional network
lncRNA: long non‐coding RNA
ROC: receiver operating characteristic
SML‐GCN: similarity‐map graph convolutional network
SVD: singular value decomposition

## 1 Introduction

Population aging is accelerating worldwide, reshaping disease burdens and straining health‐care systems (Collaborators 2020). Aging is a complex biological process driven by cumulative molecular damage, chronic inflammation, metabolic drift, and progressive loss of tissue homeostasis (J. Guo et al. 2022). These changes contribute to the onset and progression of multiple age‐related diseases, including neurodegenerative disorders (e.g., Parkinson's disease and Alzheimer's disease), metabolic and cardiovascular syndromes (e.g., type 2 diabetes mellitus, hypertension, atherosclerosis, and metabolic syndrome), as well as cerebrovascular and neuromuscular diseases (Basith et al. 2023; Y. Guo et al. 2024; Manavalan et al. 2019). Because these disorders often share overlapping biological mechanisms, identifying common and disease‐specific molecular regulators is essential for understanding aging‐related pathogenesis and developing effective biomarkers and therapeutic targets.

Long non‐coding RNAs (lncRNAs) have emerged as important regulatory molecules in aging and age‐related diseases. LncRNAs are transcripts longer than 200 nucleotides with little or no protein‐coding capacity (Kim et al. 2018). Rather than serving as passive transcriptional products, they participate in multiple layers of gene regulation, including chromatin remodeling, transcriptional control, post‐transcriptional processing, and intercellular communication (Booth and Brunet 2016). Accumulating evidence indicates that dysregulated lncRNA expression is involved in cellular senescence, inflammatory signaling, malignant transformation, neuronal dysfunction, metabolic imbalance, and immune remodeling (Ni et al. 2022; Sesma 2016; Wilson et al. 2019). These properties make lncRNAs promising candidates for biomarker discovery and therapeutic intervention in aging‐related disease contexts.

With the rapid growth of experimentally supported lncRNA‐disease association data, several curated databases have been developed to organize known associations and related biological knowledge. Representative resources include LncRNADisease (Bao et al. 2019) and MNDR (Cui et al. 2018), which provide valuable foundations for computational prediction and candidate prioritization. More broadly, recent advances in artificial intelligence and machine learning have accelerated biomedical discovery by enabling automated research workflows, biological sequence modeling, and integrative prediction from heterogeneous data sources (Basith et al. 2021; Luo et al. 2025; Y. Wang et al. 2024). Together, these advances highlight the growing importance of integrative computational frameworks for data‐driven biomedical discovery.

However, despite these advances, reliable prediction of lncRNA‐disease associations remains challenging. First, many machine‐learning models trained on limited and noisy association data exhibit poor generalization when confronted with expanding, sparser matrices; shallow or linear assumptions may fail to capture the nonlinear, context‐dependent relationships between lncRNAs and complex diseases (Basith et al. 2023; Dai et al. 2022; Manavalan et al. 2017). Second, a substantial fraction of existing predictors encodes lncRNA‐disease pairs by concatenating attributes while underutilizing the structural semantics of biological networks—i.e., information about neighborhoods, higher‐order proximity, and multiplex relationships that shape node representations (Hasan et al. 2020). Third, a methodological mismatch persists: several deep learning approaches treat associations as Euclidean, grid‐like data, despite the intrinsically graph‐structured nature of biomedical systems, where non‐Euclidean geometry better represents heterogeneous interactions and latent functional modules (Wei et al. 2014).

Graph convolutional networks (GCNs) offer a natural strategy to address these limitations by learning node representations that aggregate information from local neighborhoods under a graph Laplacian (Wu et al. 2021). Applied to well‐constructed similarity and interaction graphs, GCNs reduce noise in sparse data, transmit information through biologically relevant structures, and identify hidden association patterns. Nevertheless, effective use of GCNs for lncRNA‐disease prediction demands principled solutions on multiple levels: building and fusing heterogeneous graphs from complementary evidence, balancing linear structure with nonlinear feature learning, stabilizing optimization under severe class imbalance, and validating model contributions through rigorous ablation studies and cross‐dataset comparisons.

**FIGURE 1:**
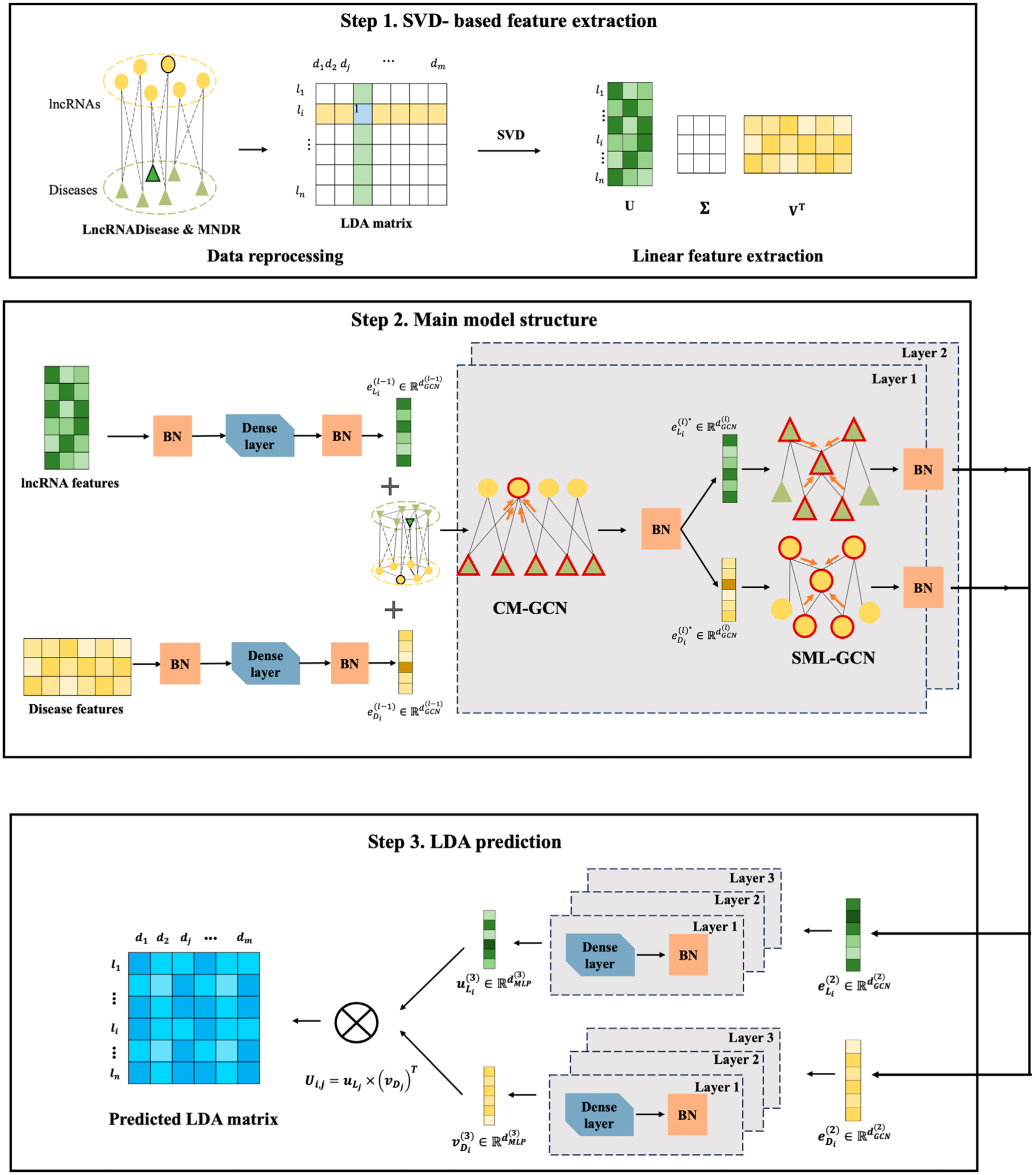
Overview of iLDA‐SGCN. The workflow of iLDA‐SGCN consists of three steps: Step 1, SVD‐based feature extraction; Step 2, construction of the main model architecture; and Step 3, LDA prediction. BN, batch normalization; CM‐GCN, correlation‐map GCN; LDA, lncRNA‐disease association; SML‐GCN, similarity‐map GCN.

Here, we introduce iLDA‐SGCN, a graph‐based framework for predicting associations between lncRNAs and age‐related diseases that integrates singular value decomposition (SVD) with a dual‐module GCN architecture (Figure 1). SVD is first applied to the lncRNA‐disease association matrix to extract compact low‐rank features that preserve dominant linear association patterns while suppressing noise. Based on these representations, iLDA‐SGCN further learns nonlinear, topology‐aware embeddings through two complementary GCN modules. The correlation‐map GCN (CM‐GCN) operates on the bipartite lncRNA‐disease interaction network to capture cross‐type relational signals, allowing disease context to refine lncRNA embeddings and vice versa. The similarity‐map GCN (SML‐GCN) operates on two homogeneous graphs, the lncRNA‐lncRNA graph and the disease‐disease graph, constructed by fusing semantic similarity (e.g., MeSH‐based disease semantics via IDSSIM) with Gaussian association profile (GAP) kernel similarity derived from the association matrix. This dual‐view design jointly exploits biological semantics and topological proximity, yielding robust multi‐view node representations.

We systematically evaluate iLDA‐SGCN against five competitive baselines—SDLDA (Zeng et al. 2020), LDNFSGB (Zhang, Ye, et al. 2020), IPCARF (R. Zhu et al. 2021), LDASR (Z. H. Guo et al. 2019), and LDA‐VGHB (Peng et al. 2023)—on two benchmark datasets, LncRNADisease and MNDR. A comprehensive protocol based on five‐fold cross‐validation and multiple metrics (AUC (area under the receiver operating characteristic curve), AUPR (area under the precision‐recall curve), precision, recall, accuracy, and F1 score) showed that iLDA‐SGCN consistently improves ranking and classification performance. Notably, the model achieved strong AUC and AUPR on MNDR and retained superior performance across most metrics on LncRNADisease, indicating a favorable balance between sensitivity and precision. Ablation analyzes further showed that each submodule contributes substantially. The full iLDA‐SGCN model surpassed all ablated variants, thereby validating the advantage of coupling linear SVD‐based structure with dual GCN components. Hyperparameter studies further clarified how epochs, learning rates, and weight decay factors shaped performance, yielding robust default settings for future applications.

Beyond cross‐validation, we conduct targeted case studies spanning aging and eight prototypical age‐related diseases (Parkinson's disease, atherosclerosis, cerebrovascular disease, type 2 diabetes mellitus, metabolic syndrome, amyotrophic lateral sclerosis, hypertension, and Alzheimer's disease). iLDA‐SGCN identifies lncRNAs with high predicted association scores, many concordant with previous studies, including *HOTAIR*, *MALAT1*, *PVT1*, *MEG3*, *H19*, *LSINCT5*, and *UCA1*—molecules with mechanistic roles in chromatin remodeling (Gupta et al. 2010), *p53* signaling (J. Zhu et al. 2015), miRNA crosstalk, telomere regulation (Jiang et al. 2020), and senescence‐associated programs (Sritharan et al. 2025).

Overall, iLDA‐SGCN advances computational discovery of lncRNA‐disease associations by (i) fusing semantic and topological evidence to build informative graphs; (ii) learning multi‐view node embeddings through a coordinated CM‐GCN and SML‐GCN design; (iii) preserving principal linear structure with SVD while enabling nonlinear refinement; and (iv) addressing sparsity and imbalance with an augmented loss. The resulting framework offers a practical, extensible tool for prioritizing lncRNAs implicated in aging and age‐related diseases and lays a methodological foundation for integrating expanding multi‐omics resources into graph‐based inference.

## 2 Results

### 2.1 Hyperparameter Selection and Experimental Settings

We systematically evaluated iLDA‐SGCN on LncRNADisease and MNDR using a hyperparameter grid with epochs (100, 500, 1000, 1500, 2000, 2500, 3000) (Huang et al. 2024), weight decay factors (0.01, 0.001), and learning rates (0.01, 0.001, 0.0001).

**FIGURE 2:**
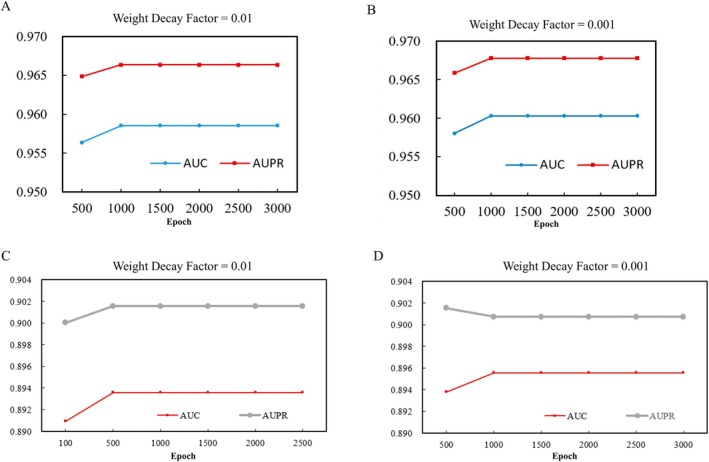
Performance of iLDA‐SGCN under different epochs and weight decay factors on LncRNADisease (A and B) and MNDR (C and D) datasets. AUC, area under the receiver operating characteristic curve; AUPR, area under the precision–recall curve.

**FIGURE 3:**
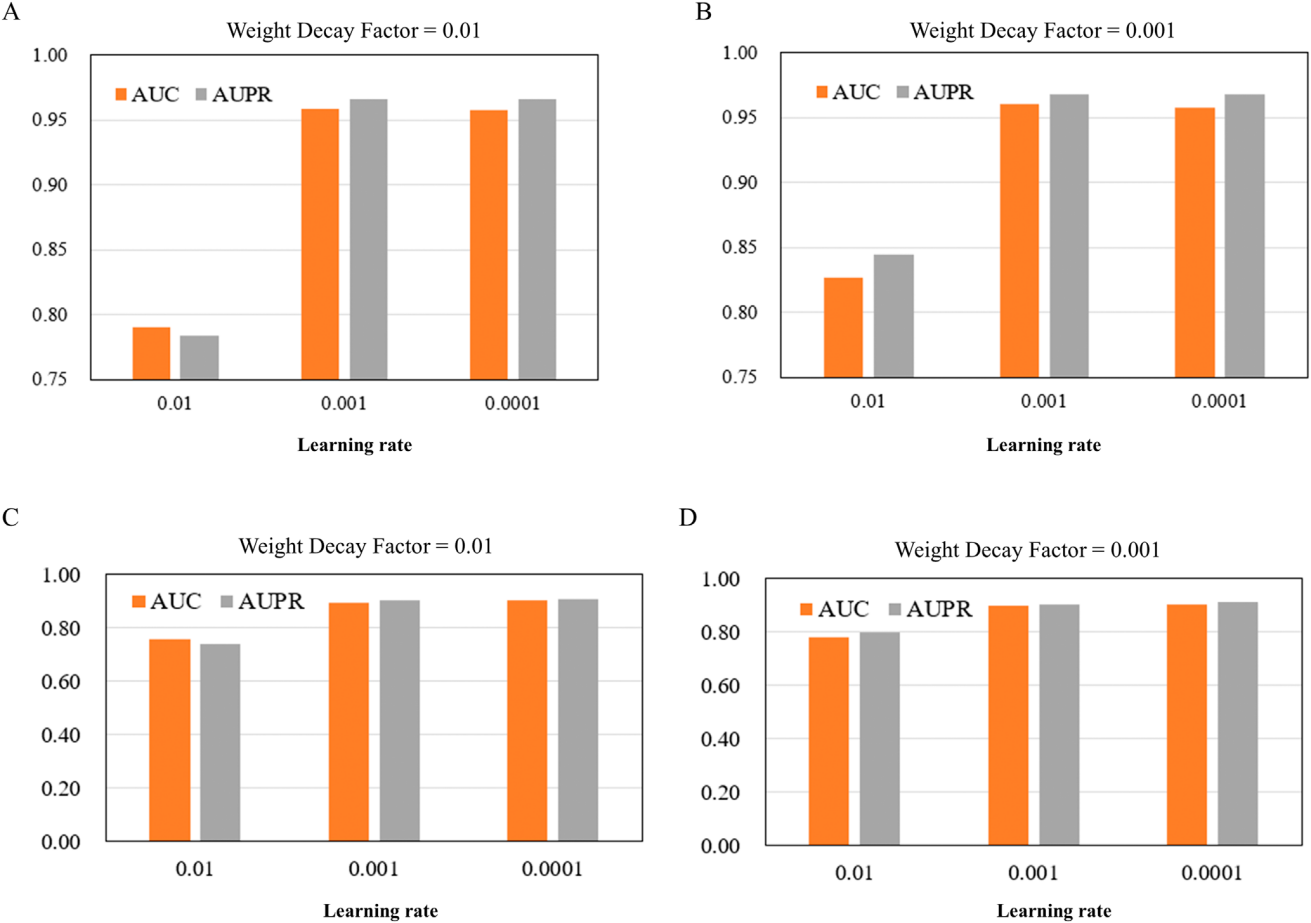
Performance of iLDA‐SGCN under different learning rates and weight decay factors on LncRNADisease (A and B) and MNDR (C and D) datasets. AUC, area under the receiver operating characteristic curve; AUPR, area under the precision–recall curve.

On the LncRNADisease dataset, a weight decay of 0.001 yielded higher AUC and AUPR than 0.01, with performance peaking at 1000 epochs (Figure 2A,B, Table S1). Similarly, on MNDR, the best overall performance was obtained with weight decay of 0.01 at 1000 epochs (Figure 2C, Table S1). By contrast, on MNDR with weight decay of 0.001, AUC was maximal at 1000 epochs, whereas AUPR was slightly lower than at 500 epochs (Figure 2D, Table S1). In addition, with a learning rate of 0.001, both AUC and AUPR were maximized regardless of whether the weight decay was 0.01 or 0.001 (Figure 3, Table S2).

Since the GCN architecture is a core component of iLDA‐SGCN, we further examined the effect of GCN depth. The results showed that a two‐layer GCN achieved the best performance, yielding the highest AUC and AUPR (Table S3). These findings suggest that the two‐layer architecture strikes a favorable balance between expressive capacity and preservation of node‐specific information, enabling it to capture complex interaction patterns while avoiding excessive smoothing.

Accordingly, considering the overall performance across both datasets and the need for a unified model configuration, we selected 1000 epochs, a learning rate of 0.001, and a weight decay of 0.001 as the final hyperparameters for iLDA‐SGCN.

### 2.2 Performance Comparison Among Different Methods

To evaluate the performance of iLDA‐SGCN, we compared it with the five representative lncRNA‐disease association prediction methods (i.e., SDLDA (Zeng et al. 2020), LDNFSGB (Zhang, Ye, et al. 2020), IPCARF (R. Zhu et al. 2021), LDASR (Z. H. Guo et al. 2019), LDA‐VGHB (Peng et al. 2023)) on the two datasets (i.e., LncRNADisease and MNDR). As summarized in Tables 1 and 2, iLDA‐SGCN achieved higher AUC and AUPR than all competing methods on both datasets. On LncRNADisease, the precision of iLDA‐SGCN was marginally lower than that of LDA‐VGHB by 0.015, whereas all remaining metrics exceeded those of LDA‐VGHB.

**TABLE 1:** Performance comparison among different methods on LncRNADisease.

	SDLDA	LDNFSGB	IPCARF	LDASR	LDA‐VGHB	iLDA‐SGCN
AUC	0.802 ± 0.048	0.735 ± 0.046	0.510 ± 0.143	0.706 ± 0.042	0.881 ± 0.042	**0.896 ± 0.012**
AUPR	0.846 ± 0.055	0.724 ± 0.063	0.534 ± 0.014	0.678 ± 0.097	0.895 ± 0.032	**0.901 ± 0.012**
Precision	0.851 ± 0.051	0.700 ± 0.064	0.488 ± 0.013	0.673 ± 0.120	**0.874 ± 0.048**	0.859 ± 0.043
Recall	0.652 ± 0.073	0.609 ± 0.079	0.572 ± 0.016	0.513 ± 0.095	0.718 ± 0.071	**0.765 ± 0.063**
ACC	0.780 ± 0.034	0.677 ± 0.042	0.491 ± 0.095	0.642 ± 0.060	0.812 ± 0.038	**0.816 ± 0.013**
F1 score	0.737 ± 0.056	0.646 ± 0.045	0.513 ± 0.011	0.567 ± 0.054	0.785 ± 0.041	**0.806 ± 0.03**

*Note:* Bold values indicate the best performance for each evaluation metric.

Abbreviations: AUC, area under the receiver operating characteristic curve; AUPR, area under the precision–recall curve; ACC, accuracy.

**TABLE 2:** Performance comparison among different methods on MNDR.

	SDLDA	LDNFSGB	IPCARF	LDASR	LDA‐VGHB	iLDA‐SGCN
AUC	0.937 ± 0.020	0.884 ± 0.027	0.711 ± 0.100	0.864 ± 0.026	0.954 ± 0.020	**0.960 ± 0.003**
AUPR	0.953 ± 0.013	0.883 ± 0.031	0.713 ± 0.101	0.867 ± 0.025	0.962 ± 0.013	**0.968 ± 0.003**
Precision	0.940 ± 0.015	0.855 ± 0.039	0.662 ± 0.097	0.841 ± 0.030	0.925 ± 0.020	**0.947 ± 0.010**
Recall	0.824 ± 0.044	0.802 ± 0.050	0.643 ± 0.015	0.736 ± 0.056	**0.860 ± 0.040**	0.83 ± 0.028
ACC	0.886 ± 0.028	0.832 ± 0.023	0.653 ± 0.078	0.797 ± 0.027	0.895 ± 0.026	**0.901 ± 0.008**
F1 score	0.878 ± 0.028	0.826 ± 0.023	0.640 ± 0.102	0.783 ± 0.026	**0.891 ± 0.023**	0.884 ± 0.017

*Note:* Bold values indicate the best performance for each evaluation metric.

Abbreviations: AUC, area under the receiver operating characteristic curve; AUPR, area under the precision–recall curve; ACC, accuracy.

### 2.3 Ablation Study

**FIGURE 4:**
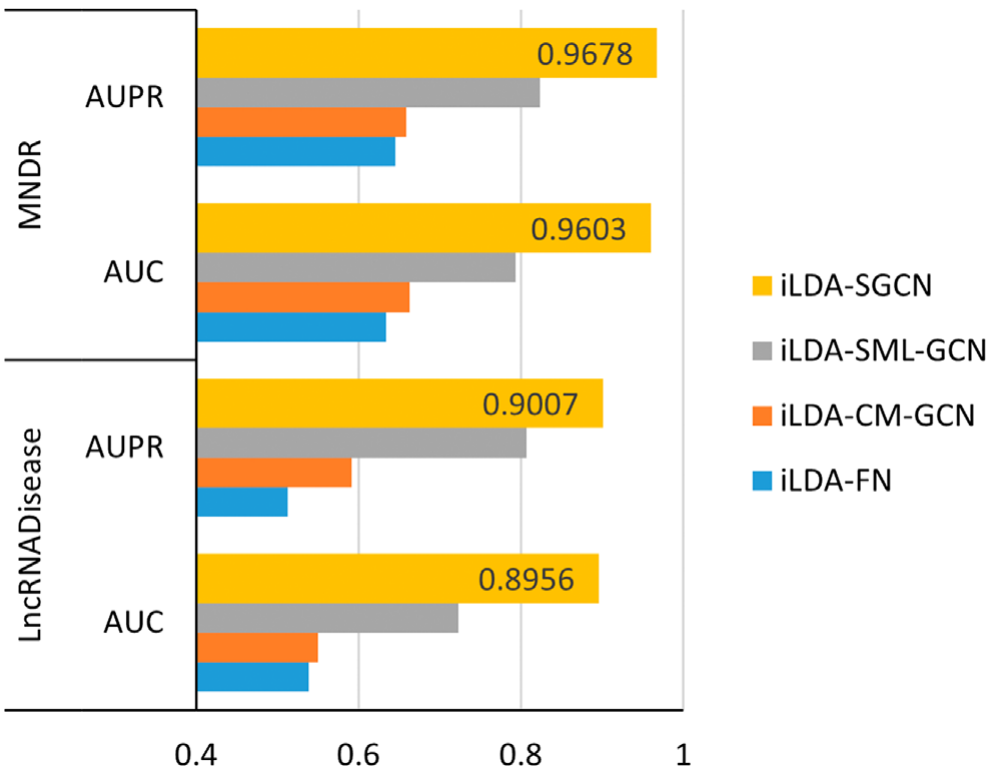
Ablation study on different models. AUC, area under the receiver operating characteristic curve; AUPR, area under the precision–recall curve.

In the iLDA‐SGCN framework, node features are extracted by three components: a fully connected network (FN), CM‐GCN, and SML‐GCN. To assess the contribution of each, we performed ablation studies on LncRNADisease and MNDR using three sub‐models: (i) iLDA‐FN (FN only), (ii) iLDA‐CM‐GCN (FN + CM‐GCN), and (iii) iLDA‐SML‐GCN (FN + SML‐GCN). Results supported the following three conclusions (Figure 4). First, both GCN‐based variants outperformed iLDA‐FN, confirming the value of graph convolution over purely dense transformations. Second, SML‐GCN delivered larger gains than CM‐GCN, indicating that leveraging homogeneous similarity graphs was particularly effective in capturing semantic structure. Third, all ablated variants underperformed the full iLDA‐SGCN, demonstrating that the combined use of FN, CM‐GCN, and SML‐GCN provided complementary information, improved representation quality, and yielded the best overall performance.

### 2.4 Case Study

These comparisons demonstrated the predictive capability of iLDA‐SGCN in prioritizing disease‐associated lncRNAs. Building on this, we further corroborated the model's top predictions with prior biological evidence and relevant literature in the context of aging and age‐related diseases, and we highlighted additional candidate lncRNAs that, to our knowledge, have not been previously reported.

This study selected aging and age‐related diseases from the MNDR database as research subjects and conducted targeted case analyzes. These eight age‐related diseases included type 2 diabetes mellitus (Fletcher et al. 2002; Palmer et al. 2019), atherosclerosis (Childs et al. 2017), hypertension (Ott and Schmieder 2022), Parkinson's disease (de Lau and Breteler 2006), cerebrovascular disease (Choi et al. 1998), amyotrophic lateral sclerosis (Xu et al. 2020), Alzheimer's disease (Scheltens et al. 2021), and metabolic syndrome (Roddy 2021).

Table 3 presents the top 10 candidate lncRNAs for aging and age‐related diseases predicted by iLDA‐SGCN on the MNDR dataset. Notably, most lncRNAs listed in the table have been supported by previous studies, indicating that the iLDA‐SGCN predictions are biologically credible. In addition, the results of iLDA‐SGCN prediction showed that *HOTAIR*, *MALAT1*, and *PVT1* were associated with up to seven age‐related diseases; *MEG3* was associated with six age‐related diseases; and *H19*, LSINCT5, and *UCA1* were associated with five age‐related diseases. LncRNAs consistently observed across multiple age‐related diseases may represent cross‐disease candidate regulators in age‐related networks and high‐priority targets for experimental validation, pointing to shared aging mechanisms across diseases. Nonetheless, potential biases should be controlled, and mechanistic and causal studies are needed to substantiate these findings. Moreover, lncRNAs without prior literature support for associations with aging or age‐related diseases likely represent biologically meaningful candidates that warrant validation in independent cohorts and functional assays.

**TABLE 3:** Top 10 candidate lncRNAs of aging and age‐related diseases predicted by iLDA‐SGCN.

Disease	Rank	lncRNA	PMID	Disease	Rank	lncRNA	PMID
Parkinson's disease	1	*TUG1*	32808542, 33085068	Type 2 diabetes mellitus	1	*MEG3*	37294471
	2	*SNHG4*	Unknown		2	*BDNF‐AS*	33360223
	3	*BANCR*	Unknown		3	*HOTAIR*	34496971
	4	*HULC*	34868009		4	*MALAT1*	33155514
	5	*SOX2‐OT*	34607512		5	*WT1‐AS*	Unknown
	6	*CASC2*	Unknown		6	*IFNG‐AS1*	Unknown
	7	*HNF1A‐AS1*	Unknown		7	*LSINCT5*	Unknown
	8	*SNHG16*	35426550		8	*SOX2‐OT*	Unknown
	9	*BCYRN1*	29850016		9	*SNHG16*	36187128
	10	*CBR3‐AS1*	Unknown		10	*DANCR*	Unknown
Atherosclerosis	1	*IGF2‐AS*	Unknown	Metabolic syndrome	1	*MALAT1*	36317460
	2	*MIAT*	34647815		2	*HOTAIR*	29922126
	3	*MALAT1*	34554676		3	*GAS5*	Unknown
	4	*MEG3*	38518516		4	*PVT1*	32454150
	5	*HOTAIR*	38250607		5	*UCA1*	Unknown
	6	*PTENP1*	Unknown		6	*H19*	32454150
	7	*PRINS*	Unknown		7	*TUG1*	34161185
	8	*PVT1*	37236049		8	*HULC*	37455763
	9	*LSINCT5*	Unknown		9	*DISC2*	Unknown
	10	*DAOA‐AS1*	Unknown		10	*CDKN2B‐AS1*	38193570
Cerebrovascular disease	1	*MALAT1*	28093478	Amyotrophic lateral sclerosis	1	*MEG3*	Unknown
	2	*GAS5*	33937403		2	*HOTAIR*	Unknown
	3	*CDKN2B‐AS1*	30870006		3	*GAS5*	Unknown
	4	*PVT1*	34521353		4	*PVT1*	39406341
	5	*H19*	34058282		5	*UCA1*	Unknown
	6	*BDNF‐AS*	Unknown		6	*H19*	39406341
	7	*SNHG16*	35320391		7	*TUG1*	34356873
	8	*WT1‐AS*	Unknown		8	*HULC*	Unknown
	9	*IFNG‐AS1*	Unknown		9	*WT1‐AS*	Unknown
	10	*UCA1*	33458871		10	*CDKN2B‐AS1*	Unknown
Hypertension	1	*H19*	32698630	Aging	1	*IGF2‐AS*	38017373
	2	*MALAT1*	38010958		2	*HOTAIR*	32489713
	3	*MIAT*	35155877		3	*GAS5*	34711157
	4	*HOTAIR*	Unknown		4	*MALAT1*	37385572
	5	*HIF1A‐AS1*	Unknown		5	*WRAP53*	Unknown
	6	*MEG3*	35995213		6	*MKRN3‐AS1*	Unknown
	7	*LSINCT5*	Unknown		7	*PVT1*	Unknown
	8	*BCYRN1*	Unknown		8	*MINA*	Unknown
	9	*PVT1*	35036445		9	*MEG3*	38518516
	10	*UCA1*	31355277		10	*H19*	33193379
Alzheimer's disease	1	*TUG1*	38490634				
	2	*PTENP1*	Unknown				
	3	*LSINCT5*	Unknown				
	4	*MINA*	Unknown				
	5	*HULC*	Unknown				
	6	*CCAT1*	Unknown				
	7	*SPRY4‐IT1*	Unknown				
	8	*SRA1*	21271304				
	9	*DGCR5*	Unknown				
	10	*PRINS*	Unknown				

In summary, iLDA‐SGCN identified 33 candidate lncRNAs potentially associated with aging and age‐related diseases; some are supported by prior studies, whereas others may represent novel candidates. These findings further substantiate the reliability of iLDA‐SGCN for lncRNA discovery and provide testable molecular candidates for subsequent mechanistic investigation and therapeutic exploration in aging biology.

## 3 Discussion

In this study, we developed a deep learning model, iLDA‐SGCN, that uses singular value decomposition and graph convolutional networks to predict unknown associations between lncRNAs and age‐related diseases. On the one hand, we used two biological databases (LncRNADisease and MNDR) to obtain associations between lncRNAs and diseases, to construct lncRNA similarity matrices and disease similarity matrices, and then to construct lncRNA‐disease association networks. On the other hand, the two modules, CM‐GCN and SML‐GCN, were designed to capture graph structure information and hidden association patterns, thus effectively predicting lncRNAs that are potentially associated with age‐related diseases.

To assess effectiveness, we benchmarked iLDA‐SGCN against representative methods on LncRNADisease and MNDR. Across both datasets, iLDA‐SGCN delivered higher AUC and AUPR than competing approaches. On MNDR, the model achieved AUC 0.960 and AUPR 0.968; on LncRNADisease, it attained AUC 0.896 and AUPR 0.901. The results suggested that iLDA‐SGCN prioritizes lncRNA‐disease links with high classification and ranking performance.

In the case study, iLDA‐SGCN identified the top 10 ranked lncRNAs with potential associations to aging and age‐related diseases. First, we examined literature support for prioritized lncRNAs identified by iLDA‐SGCN that are associated with aging and age‐related diseases. Results showed that *HOTAIR*, *MALAT1* and *PVT1* were linked to as many as seven age‐related diseases. Among these, *HOTAIR* (i.e., HOX antisense intergenic RNA) has been demonstrated to directly regulate cellular senescence. *HOTAIR* recruits the PRC2 complex to catalyze the formation of repressive *H3K27me3* marks, thereby silencing the *CDKN2A* locus containing key senescence genes such as *p16INK4a*. During aging, dysregulation of *HOTAIR* disrupts normal gene regulation, potentially leading to indirect accumulation of *p16INK4a* protein and ultimately triggering cellular senescence (Gupta et al. 2010). Furthermore, *HOTAIR* is typically upregulated during aging, disrupting stem cell self‐renewal and causing stem cell exhaustion (Gupta et al. 2010). *MALAT1* delays cellular senescence by inhibiting miR‐22, a pro‐senescence miRNA (Lettieri‐Barbato et al. 2022). During aging, *MALAT1* downregulation releases miR‐22 inhibition, promoting cell cycle arrest. Furthermore, it interacts with deacetylase SIRT1 and other factors to influence stress responses and cell survival (Kim et al. 2018; Ni et al. 2022). *PVT1* primarily functions as a pro‐senescence factor (Li et al. 2021). Its upregulation represents a key molecular event driving multiple cell types into senescence and accelerating the progression of various age‐related diseases (Prattichizzo et al. 2016).

*MEG3* is associated with six age‐related diseases. Although *MEG3* does not encode a protein, it can directly interact with the *p53* protein, promoting its stability and transcriptional activity. *MEG3* expression upregulates *p53* downstream target genes, particularly *p21* (*CDKN1A*), a potent cell cycle inhibitor that forces cells to exit the cell cycle and enter senescence. Additionally, *H19*, *LSINCT5*, and *UCA1* are associated with five age‐related diseases. *H19* exerts pro‐aging effects by serving as a source of miR‐675, while also acting as a sponge for other miRNAs to exert anti‐aging effects (Zhang, Jiang, et al. 2020). Alterations in *H19* expression can influence telomerase activity and telomere length.

Furthermore, *H19* participates in regulating the DNA damage response, and its dysfunction may lead to increased genomic instability, thereby accelerating aging (Zhao et al. 2023). *LSINCT5* expression levels directly influence cellular proliferation capacity, a core hallmark of aging (Sritharan et al. 2025). *UCA1* is associated with aging. It influences the aging process by regulating autophagy, inhibiting apoptosis, and inducing the senescence‐associated secretory phenotype (SASP) (Ghanam et al. 2017; Puvvula 2019). These findings align with the iLDA‐SGCN identification results, providing literature‐based support for the biological plausibility of the predictions.

Then, regarding lncRNAs currently lacking explicit literature reports on their association with aging and age‐related diseases, this study posits that they may represent potential candidate molecules. For instance, although direct evidence remains insufficient for lncRNAs such as *WRAP53*, *MKRN3‐AS1*, and *MINA*, their known biological functions suggest potential relevance to the aging process, warranting further investigation.

Nevertheless, several limitations should be acknowledged. First, iLDA‐SGCN relies on curated lncRNA‐disease association databases, which may contain reporting bias and incomplete annotations. Second, unobserved lncRNA‐disease pairs were treated as negative samples during model evaluation, although some of them may represent undiscovered positive associations. Third, the case‐study evidence was mainly based on literature support and computational prioritization rather than direct experimental validation. Future studies should validate these predicted associations in independent cohorts, disease‐relevant cellular or animal models, and functional assays. Incorporating tissue‐specific expression profiles, single‐cell data, and other multi‐omics resources may further improve the biological interpretability and predictive robustness of iLDA‐SGCN.

In summary, iLDA‐SGCN can effectively identify lncRNAs that are potentially associated with age‐related diseases. It reveals links between specific lncRNAs and a wide range of aging and age‐related diseases. These insights may be of potential value for early diagnosis, monitoring of therapeutic response, and the development of new therapeutics.

## 4 Materials and Methods

### 4.1 Data Sources

We collected data from two lncRNA‐disease association databases, LncRNADisease (v2.0) and MNDR (2.0), for potential lncRNA identification (Table 4). In the following, they are denoted as LncRNADisease and MNDR, respectively. LncRNADisease is a comprehensive database that provides a multitude of human LDAs for 166 diseases (Bao et al. 2019). MNDR is a comprehensive database that encompasses over 260,000 non‐coding RNA‐disease associations, systematically organized under a unified framework (Cui et al. 2018). The two databases provide valuable resources for the analysis of disease mechanisms and the design of therapeutic strategies. It is important to note that diseases lacking MeSH information or those with irregular names were excluded from the present study. Furthermore, we excluded lncRNAs without sequence data.

**TABLE 4:** The information of two lncRNA‐disease association datasets.

Datasets	lncRNA	Disease	lncRNA‐disease association
LncRNADisease	82	157	605
MNDR	89	190	1529

As defined in Equation (1), if the i‐th lncRNA $l_{i}$ is associated with disease $d_{j}$, we set $N_{\mathrm{ij}}$ to 1, otherwise, the value is 0:

(1) $$N_{\mathrm{ij}}=\left\{\begin{array}{c} 1,\text{if lncRNA}\;l_{i}\;\text{is associated with disease}\;d_{j}\\ 0,\;\text{otherwise} \end{array}\right.$$

### 4.2 Similarity Computation

To assess disease similarity, we computed the semantic similarity matrix ${\mathrm{Sim}}_{d}^{\mathrm{sem}}$ between diseases using the IDSSIM model based on their MeSH descriptions. The lack of directed acyclic graphs for some diseases in the MeSH database makes it impossible to calculate the semantic similarity between them. Therefore, we utilized the Gaussian Association Profile (GAP) kernel similarity (D. Wang et al. 2010) to calculate the semantic similarity between these diseases. The formula for calculating the kernel similarity is shown in Equation (2), where $N_{.i}$ and $N_{.j}$ are the GAPs of the two diseases $d_{i}$ and $d_{j}$, corresponding to the i‐th and j‐th columns of N, respectively.

(2) $${\text{GSim}}_{d}\left(i,j\right)=e^{-\theta_{d}∥N_{.i}-N_{.j}{∥}^{2}}$$

$\theta_{d}$ is denoted as:

(3) $$\theta_{d}=\frac{1}{m}\sum_{i=1}^{m}∥N_{.i}{∥}^{2}$$

Semantic similarity captures disease similarity from a biological perspective, while GAP‐kernel similarity quantifies proximity in the network topology. The disease similarity matrix ${\mathrm{Sim}}_{d}$ is derived by fusing these two measures. The calculation formula is shown in Equation (4).

(4) $${\mathrm{Sim}}_{d}=w\times {\mathrm{Sim}}_{d}^{\mathrm{sem}}+\left(1-w\right)\times {\text{GSim}}_{d}$$

Analogously, we derived lncRNA functional similarity by combining an IDSSIM‐based measure (via disease semantics) with a GAP kernel computed from the association matrix:

(5) $${\text{GSim}}_{l}\left(i,j\right)=e^{-\theta_{l}∥N_{.i}-N_{.j}{∥}^{2}}$$

$\theta_{l}$ is denoted as:

(6) $$\theta_{l}=\frac{1}{n}\sum_{i=1}^{n}∥N_{.i}{∥}^{2}$$

lncRNA functional similarity matrix ${\mathrm{Sim}}_{l}$ is calculated as follows:

(7) $${\mathrm{Sim}}_{l}=w\times {\mathrm{Sim}}_{l}^{\mathrm{sem}}+\left(1-w\right)\times {\text{GSim}}_{l}$$

### 4.3 Feature Extraction

Singular Value Decomposition (SVD) is a generalization of feature decomposition, which can be used to extract features efficiently through feature decomposition, and the method has been widely used for feature extraction. By selecting larger singular values, SVD reduces the dimensionality of the data and removes features that have little effect on the variability of the data, thus reducing the storage and computational cost of the data. Feature vectors with smaller singular values represent noisy or redundant parts of the data. By selecting larger singular values, SVD can retain the main linear features, thus removing the noisy and redundant parts of the data.

We applied SVD to extract linear features of diseases and lncRNAs. SVD decomposed the lncRNA‐disease association matrix Y into two orthogonal matrices and one diagonal matrix by feature decomposition, as shown in Equation (8), where $U\in {\mathbb{R}}^{n\times n}$ and $V\in {\mathbb{R}}^{m\times m}$ are two real matrices. $V^{T}$ is the matrix transpose of *V*. Σ is a diagonal matrix consisting of singular values n.

(8) $$Y=U\Sigma V^{T}$$

The top q singular values are approximated using Equation (9), where $U_{i}$ represents the representation of the i‐th lncRNA $l_{i}$ and ${V_{j}}^{T}$ represents the characterization of the *j*‐th disease. In this study, the number of retained singular values was set to 32.

(9) $$R\approx U_{i}\sum_{q}{V_{j}}^{T}$$

### 4.4 Node Feature Extraction Based on GCN

GCN‐based node representation is a key step in identifying lncRNAs and disease associations. GCNs aggregate information about neighboring nodes and capture hidden network structures, allowing for robust extraction of node features. Therefore, we use GCN to learn node features of lncRNAs and diseases from heterogeneous lncRNA‐disease association networks. Let $e^{l}\in {\mathbb{R}}^{d}$ denote the node embedding at the *l*‐th layer of the GCN. The node embedding at the subsequent layer, $e^{l+1}\in {\mathbb{R}}^{d}$, is computed according to Equations ((10), (11), (12)), where *S* is the adjacency matrix, *I* is the identity matrix, and $\tilde{D}$ is the diagonal degree matrix of $\tilde{S}$. Each diagonal element of $\tilde{D}$ corresponds to the degree of a node. $W^{l}$ denotes the trainable weight matrix at layer *l*, and $\sigma \left(\cdot \right)$ is the activation function.

(10) $$e^{l+1}=\sigma \left({\tilde{D}}^{-\frac{1}{2}}\times {\tilde{S}\tilde{D}}^{-\frac{1}{2}}\times e^{l}W^{l}\right)$$

(11) $$\tilde{S}=I+S$$

(12) $$\tilde{D}\left(i,i\right)=\sum_{j}\tilde{S}\left(i,j\right)$$

The model consists of two main core modules: the CM‐GCN (correlation‐map graph convolutional network) and the SML‐GCN (similarity‐map graph convolutional network). In our implementation, both CM‐GCN and SML‐GCN adopted a two‐layer GCN architecture. These modules were designed to learn node representations from distinct subnetworks of the heterogeneous lncRNA‐disease association network, thereby capturing complementary relational and semantic structure in a coordinated manner.

The CM‐GCN module was utilized to aggregate node information from the lncRNA‐disease interaction network, enabling it to capture hidden association features among heterogeneous nodes. This module was able to reveal potential heterogeneous association features between lncRNAs and disease nodes that are critical for understanding the role of lncRNAs in disease. We denoted this graph by $G_{\mathrm{CM}}=\left\{{\mathrm{ND}}_{d},{\mathrm{ND}}_{l},A_{d-l}\right\}$, where ${\mathrm{ND}}_{d}$ and ${\mathrm{ND}}_{l}$ represent the disease nodes and lncRNA nodes, respectively, and $A_{d-l}$ encodes lncRNA‐disease interactions. Within this module, lncRNA representations were updated by message passing from disease neighbors, and disease representations were analogously updated from lncRNA neighbors, thereby revealing heterogeneous association patterns that are informative for disease mechanisms.

The node embeddings produced by CM‐GCN were then fed into SML‐GCN to further exploit semantic structure on two homogeneous similarity graphs: the lncRNA‐lncRNA similarity graph $G_{l}=\left\{{\mathrm{ND}}_{l},A_{l-l}\right\}$ and the disease‐disease similarity graph $G_{d}=\left\{{\mathrm{ND}}_{d},A_{d-d}\right\}$. $G_{l}$ and $G_{d}$ are the two main inputs of SML‐GCN. SML‐GCN refined lncRNA (or disease) embeddings by aggregating information from their homogeneous neighbors, thus enhancing the model's capacity to encode functional similarity and higher‐order semantics in the lncRNA‐disease space.

Batch normalization was applied after each deep module to reduce internal covariate shift and improve optimization stability. With this two‐stage design, iLDA‐SGCN learned from both direct cross‐type interactions and homogeneous semantic neighborhoods, enabling comprehensive integration of structural and semantic information for lncRNA‐disease association inference.

### 4.5 Association Prediction

To further eliminate redundancy and noise, three consecutive fully connected layers were designed in this study to extract high‐level node features. Each layer has 400, 200, and 100 neurons, respectively.

Assume the GCN modules output embedded $h_{L_{i}}$ for lncRNA nodes and $h_{D_{j}}$ for disease nodes. These embeddings were then refined by a three‐layer MLP, yielding the final representation $u_{L_{i}}$ and $v_{D_{j}}$. Association scores were computed with an inner‐product decoder (Equation 13), producing the prediction matrix U. Larger $U_{i,j}$ values indicate a higher predicted likelihood of association between lncRNA $L_{i}$ and disease $D_{j}$.

(13) $$U_{i,j}=u_{L_{i}}\times {\left(v_{D_{j}}\right)}^{T}$$

We first adopted a mean‐squared error (MSE) objective that minimized the Frobenius‐norm discrepancy between the predicted score matrix U and the label matrix $B_{d}$. However, the number of negative associations is substantially higher than that of positive associations, which can bias the MSE loss toward negatives. To alleviate this class imbalance, we employed a υ‐enhanced loss function designed to assign greater weight to positive samples (Equation 14). Specifically, ${\tilde{B}}_{d}$ is the enhanced association matrix constructed from the original adjacency matrix $B_{d}$. υ is the hyperparameter that determines the margin between true labels and predicted scores. μ is the weight‐decay coefficient used to regularize the trainable parameters W and reduce overfitting. U is the score matrix predicted by iLDA‐SGCN. The loss function is defined as:

(14) $$\text{Loss}={∥{\tilde{B}}_{d}-U∥}_{F}^{2}+\mu {∥W∥}_{2}^{2}$$

The enhanced label matrix ${\tilde{B}}_{d}$ is given by:

(15) $${\tilde{B}}_{d}=\left\{\begin{array}{c} 0,\text{if}\;B_{d}\left(i,j\right)=0\;\text{or}\;B_{d}\left(i,j\right)\in D_{\mathrm{ind}}\\ \upsilon ,\text{otherwise} \end{array}\right.$$

### 4.6 Performance Evaluation

To evaluate the effectiveness of iLDA‐SGCN, we performed five‐fold cross‐validation on two benchmark datasets, LncRNADisease and MNDR. The evaluation was conducted at the association level, i.e., each sample corresponds to a lncRNA–disease pair. Experimentally verified lncRNA–disease associations were treated as positive samples, whereas unobserved pairs were treated as negative samples. To alleviate class imbalance, we randomly sampled an equal number of negative pairs from the unobserved set, resulting in a balanced dataset (positive: negative = 1:1).

The positive and negative sets were then separately shuffled and split into five folds. In each run, four folds from each set were used for training, and the remaining fold was used for testing. Importantly, only the training‐fold positive associations were used to construct the relation matrix for graph propagation, while all test‐fold associations were strictly held out. This procedure was repeated across all five folds, and the final results were reported as the average performance over the five runs. To ensure reproducibility, we fixed the random seeds for NumPy and PyTorch to 666.

Model performance was assessed using six standard metrics: AUC, AUPR, Precision, Recall, Accuracy, and F1‐score. The corresponding definitions are provided in Equations ((16), (17), (18), (19), (20)).

(16) $$F1\;\text{score}=\frac{2\times \text{Precision}\times \text{Recall}}{\text{Precision}+\text{Recall}}$$

(17) $$\mathrm{ACC}=\frac{\mathrm{TP}+\mathrm{TN}}{\mathrm{TP}+\mathrm{FP}+\mathrm{TN}+\mathrm{FN}}$$

(18) $$\text{Presion}=\frac{\mathrm{TP}}{\mathrm{TP}+\mathrm{FP}}$$

(19) $$\text{Recall}=\frac{\mathrm{TP}}{\mathrm{TP}+\mathrm{FN}}$$

(20) $$\mathrm{FPR}=\frac{\mathrm{FP}}{\mathrm{FP}+\mathrm{TN}}$$

Here, TP denotes the number of true positives (samples correctly predicted as positive), TN the number of true negatives (samples correctly predicted as negative), FP the number of false positives (samples incorrectly predicted as positive), and FN the number of false negatives (samples incorrectly predicted as negative). AUC is the area under the receiver operating characteristic (ROC) curve plotting the true‐positive rate (TPR) against the false‐positive rate (FPR). AUPR is the area under the precision‐recall curve and is particularly informative when classes are highly imbalanced.

## Author Contributions

Y.G. designed research; Y.G. conducted research; Y.G. analyzed data and performed statistical analysis; Y.G. wrote the paper. S.Q., Z.Z., J.G., H.L., and H.Y. helped proofread the manuscript. Y.H., F.L., and Y.X. provided important advice for this manuscript. Y.H. had primary responsibility for the final content. All authors read and approved the final manuscript.

## Funding

This work was supported by the National Natural Science Foundation of China (No: 62371161), 0–1 Original Exploration Category: Fundamental Research Funds for the Central Universities Project (No. 2022FRFK030025), Heilongjiang Provincial Science and Technology Tackling Project (No. GNCMSSJH2024), and Research and Innovation Fund of The First Affiliated Hospital of Harbin Medical University (2021M13).

## Conflicts of Interest

All authors declare no competing interests.

## Supporting information


**Table S1:** Performances of the parameters Epoch and weight decay factor on LncRNADisease, and MNDR.
**Table S2:** Performances of the parameters learning rate and weight decay factor on LncRNADisease and MNDR.
**Table S3:** The impact of GCN layers on the predictive performance of iLDA‐SGCN.

## Data Availability

The code and datasets used in this study can be accessed at https://github.com/yyy‐nkl/iLDA‐SGCN. Specifically, the model was implemented and run under Python 3.7, with the following core packages: tensorflow‐gpu 2.6.0, NumPy 1.19.5, pandas 1.1.5, and scikit‐learn 0.24.2.
